# Clusters of Basic Amino Acids Contribute to RNA Binding and Nucleolar Localization of Ribosomal Protein L22

**DOI:** 10.1371/journal.pone.0005306

**Published:** 2009-04-23

**Authors:** Jennifer L. Houmani, Ingrid K. Ruf

**Affiliations:** Department of Molecular Biology and Biochemistry, University of California Irvine, Irvine, California, United States of America; University of Hong Kong, Hong Kong

## Abstract

The ribosomal protein L22 is a component of the 60S eukaryotic ribosomal subunit. As an RNA-binding protein, it has been shown to interact with both cellular and viral RNAs including 28S rRNA and the Epstein-Barr virus encoded RNA, EBER-1. L22 is localized to the cell nucleus where it accumulates in nucleoli. Although previous studies demonstrated that a specific amino acid sequence is required for nucleolar localization, the RNA-binding domain has not been identified. Here, we investigated the hypothesis that the nucleolar accumulation of L22 is linked to its ability to bind RNA. To address this hypothesis, mutated L22 proteins were generated to assess the contribution of specific amino acids to RNA binding and protein localization. Using RNA-protein binding assays, we demonstrate that basic amino acids 80–93 are required for high affinity binding of 28S rRNA and EBER-1 by L22. Fluorescence localization studies using GFP-tagged mutated L22 proteins further reveal that basic amino acids 80–93 are critical for nucleolar accumulation and for incorporation into ribosomes. Our data support the growing consensus that the nucleolar accumulation of ribosomal proteins may not be mediated by a defined localization signal, but rather by specific interaction with established nucleolar components such as rRNA.

## Introduction

Assembly of eukaryotic ribosomal subunits occurs in the cell nucleolus where ribosomal proteins are assembled along with rRNA by a myriad of processing and assembly factors (reviewed in: [1]). The nucleolus is a dynamic structure, breaking down during mitosis and reassembling around centers of rDNA transcription following cell division [2]. Ribosomal proteins, which like other proteins are translated in the cytoplasm, must be imported into the nucleus via an active transport mechanism mediated by a nuclear localization signal (NLS) and then transit to the nucleolus. While many nucleolar proteins contain classical monopartite or bipartite NLSs [3], [4], Stuger, et al. proposed that eukaryotic ribosomal proteins utilize a unique nuclear import pathway mediated by a novel consensus NLS [5]. In contrast to nuclear import, the mechanism by which ribosomal proteins accumulate in the nucleolus is not well understood. A number of retroviral proteins are known to contain a specific nucleolar targeting signal composed of basic amino acid clusters, however this consensus sequence is not generally found in cellular nucleolar proteins [6]. Because the nucleolus is not a membrane-bound structure, it is presumed that nucleolar accumulation occurs via interaction with established nucleolar components such as rRNA [2]. While a number of studies have examined the sequence requirements for the nucleolar localization of ribosomal proteins [7]–[13], relatively few have examined rRNA binding as a means for nucleolar accumulation [14]–[17].

The ribosomal protein L22, a component of the 60S ribosomal subunit, has been characterized as an RNA-binding protein. Early studies of L22 termed the protein EAP for EBER-associated protein in reference to its interaction with a small viral RNA encoded by Epstein-Barr virus (EBV) [18], [19]. L22 is unique to eukaryotes and its cellular function has yet to be clearly defined. Studies demonstrating that partially reconstituted ribosomes lacking L22 are active for translation *in vitro* suggest that L22 may function in a regulatory capacity and have extra-ribosomal functions [20]. This is supported by recent evidence that germline disruption of the *RPL*22 gene in mice is not lethal whereas this is the case for other ribosomal proteins [21], [22]. L22 has been observed to bind both cellular and viral RNAs [18], [19], [23]–[25]. Its interaction with the EBV-encoded RNA EBER-1 has been well characterized by mutational analyses which reveal that point mutations in the base paired nucleotides of the stem as well as nucleotides in the loop of EBER-1 stem-loop III significantly decrease binding by L22 *in vitro* [19]. Subsequent studies have shown that L22 can bind three sites on EBER-1 encompassing portions of stem-loops I, III and IV [18], [19], [26], [27]. The most frequently isolated cellular RNA sequence bound by L22 maps to stem-loop 7 of 28S rRNA [25], [28]. Additional regions of 28S rRNA, as well as regions of 18S rRNA, have also been shown to interact with L22 *in vitro* [25]. Comparison of RNA sequences bound by L22 has allowed for the establishment of a consensus L22 binding site consisting of a stem-loop structure with a G-C base pair at the base of the loop and a 5–7 nucleotide loop with a U residue at the 3′ end [25].

Although the accumulation of L22 in nucleoli has been demonstrated and a specific amino acid sequence has been shown to contribute to nucleolar localization [29], an RNA-binding domain has not been defined, nor has a link between rRNA binding and nucleolar accumulation been established. Here, we investigated the sequences required for RNA binding and nucleolar localization of L22 using RNA-binding assays and fluorescence localization studies. We demonstrate that a specific cluster of basic amino acids is critical for high affinity RNA binding and for the nucleolar accumulation of L22, thereby linking rRNA binding to nucleolar accumulation of this protein.

## Results

### L22 binds EBER-1 and 28S rRNA *in vivo*

**Figure 1:**
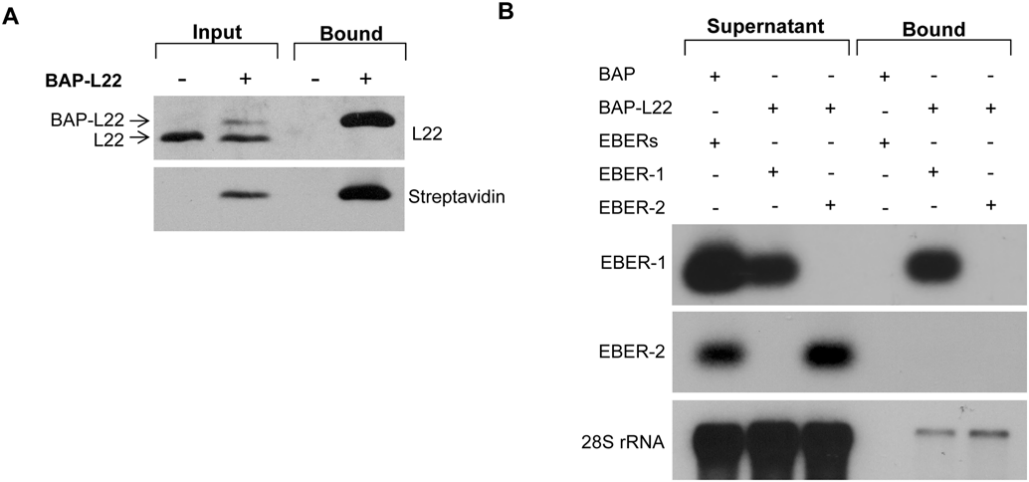
L22 binds EBER-1 and 28S rRNA *in vivo*. (A) Expression and biotinylation of BAP-L22 in transiently transfected 293T cells. Transfected cells were lysed and expression of BAP-L22 was verified by immunoblot with anti-L22 antibody (top panel). Successful biotinylation was confirmed by immunoblot with HRP-conjugated streptavidin (bottom panel). Specific capture of biotinylated L22 was demonstrated by immunoblot analysis following incubation of protein lysates with avidin agarose and stringent washing (bound). (B) Specific binding of EBER-1 and 28S rRNA by BAP-L22. 293T cells were transiently co-transfected with either BAP or BAP-L22 and EBERs, EBER-1, or EBER-2 as indicated. 48 hrs post-transfection, cells were UV crosslinked and biotinylated L22 was isolated using avidin agarose, as above. RNA was isolated from both the supernatant and pellet of affinity capture reactions. 2.5 µg RNA from each supernatant sample along with entire RNA sample from each pellet was analyzed by northern hybridization using probes specific for EBER-1, EBER-2, and 28S rRNA, as indicated.

To confirm previous reports [19], [25]–[27] and establish that L22 interacts with both EBER-1 and 28S rRNA *in vivo*, we utilized a biotin-avidin affinity assay to isolate L22 and any associated RNA. In this assay, a 17-amino acid biotin acceptor peptide (BAP) was fused to the N-terminus of L22 and biotinylation was accomplished *in vivo* using a co-expressed bacterial biotin ligase (BirA). 293T cells were transiently co-transfected with expression constructs encoding BAP or BAP-L22, BirA, and EBER-1, EBER-2 or both EBERs. Following UV crosslinking and lysis of cells, biotinylated proteins were captured on avidin beads and analyzed by immunoblot for the presence of L22 (Fig. 1A, top panel) and biotin (Fig. 1A, lower panel). After confirming that BAP-L22 was efficiently biotinylated and successfully captured on avidin beads, RNA associated with the isolated proteins was extracted and detected by northern blot analysis. As shown in Fig. 1B, while EBER-1 (lane 5) and 28S rRNA (lanes 5–6) were isolated along with biotinylated BAP-L22, EBER-2 (lane 6) was not isolated. Furthermore, none of these RNAs were isolated in the presence of only BAP (lane 4) demonstrating that the observed binding was specific for L22 and not an artifact of the BAP tag. These results clearly demonstrate that L22 binds strongly to full-length EBER-1 and, to a lesser extent, endogenous 28S rRNA *in vivo*.

### Clusters of basic amino acids are required for L22 to bind to RNA

**Figure 2:**
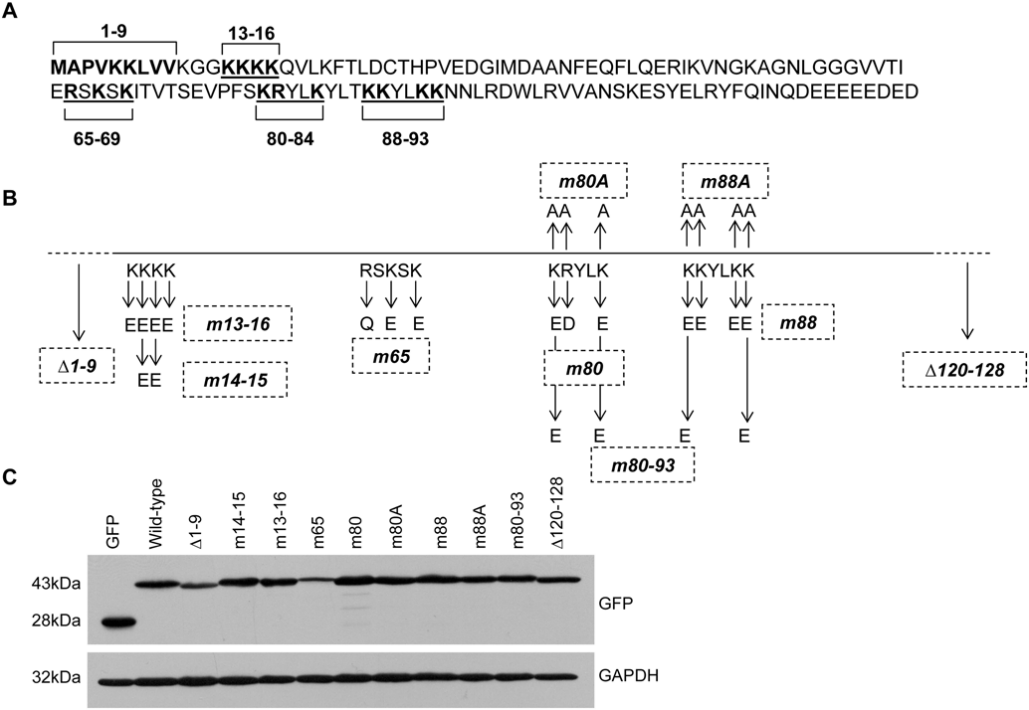
Generation and characterization of wild-type and mutated L22 expression constructs. (A) Clusters of basic amino acids likely to be involved in RNA binding were identified within the L22 amino acid sequence and are shown underlined with the basic residues highlighted in bold font. The nine amino-terminal residues previously predicted to be the RNA-binding domain are also highlighted in bold [29]. (B) Location of mutations introduced into L22 coding sequence. Constructs expressing L22 lacking either nine amino-terminal or eight carboxy-terminal residues are designated Δ1–9 and Δ120–128, respectively. Point mutations generated in the basic amino acid clusters illustrated in (A) are shown relative to the wild-type sequence (shown directly below the line) and designated by arrows above and below the line. For m80 and m88, constructs with K to E mutations (and R to D) have been designated m80 and m88 while constructs with K to A mutations have been designated m80A and m88A. (C) N-terminal GFP-L22 fusion constructs, depicted in (B), were transiently transfected into 293T cells followed by analysis of protein lysates for protein expression by immunoblot using anti-GFP antibody. GAPDH served as a control for protein loading.

To define the amino acids of L22 required for RNA binding, we generated a series of N-terminally fused GFP-L22 expression constructs in which regions of L22 likely to be involved in RNA binding were mutated (Fig. 2A). These mutations included several clusters of basic amino acids chosen largely based on the prediction that positively charged amino acids are likely to interact with negatively charged nucleic acids. As depicted in Fig. 2B, select lysine and arginine residues were mutated to glutamic acid and aspartic acid residues or to alanine residues. Furthermore, based on previous work by Shu-Nu *et al.* [29], two additional constructs were generated in which either the N-terminus (Δ1–9) or C-terminus (Δ120–128) was truncated. Protein expression from each construct was confirmed following transient transfection into 293T cells and subsequent immunoblot analysis (Fig. 2C). While proteins of the predicted size were efficiently expressed from the majority of constructs, m65 was consistently expressed at reduced levels relative to wild-type L22 and the other mutated L22 proteins. This is likely the result of reduced transfection efficiency using this construct or potentially indicative of cellular cytotoxicity or protein misfolding.

**Figure 3:**
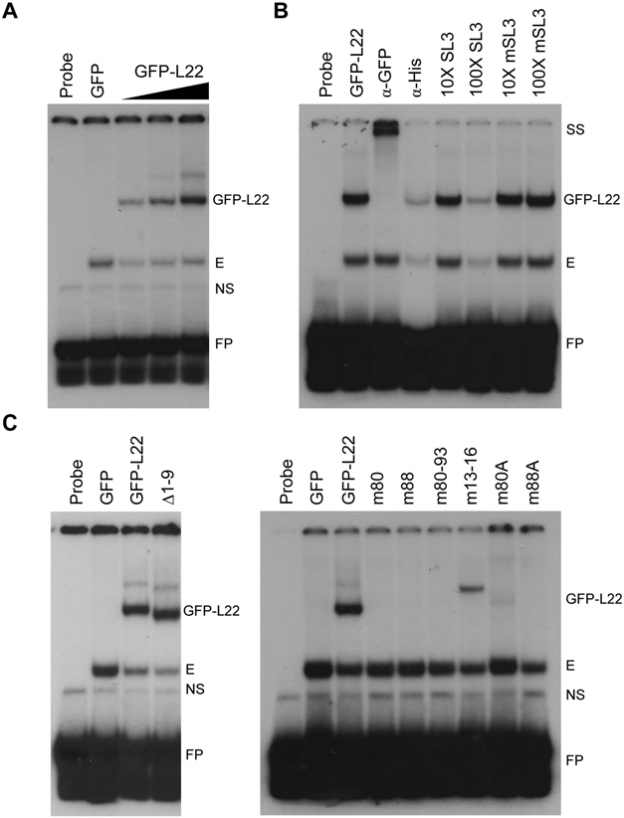
Clusters of basic amino acids mediate L22 binding to EBER-1. (A) Binding of GFP-L22 to stem-loop III of EBER-1 (SL3) was tested in RNA EMSA experiments using increasing amounts of protein lysate (1, 5, and 20 µg) generated from 293T cells transfected with GFP-L22. 5 µg of control lysate expressing only GFP was used to assess nonspecific binding. Each 10 µl binding reaction contained 0.05 pmoles ^32^P end-labeled SL3 RNA oligonucleotide. Reactions were electrophoresed on 8% native polyacrylamide gels and visualized by autoradiography. (B) L22 binds specifically to EBER-1. Binding specificity of L22 to SL3 was tested by antibody supershift and by competition with unlabeled oligonucleotides. 5 µg of GFP-L22 protein lysate was used in binding reactions. For antibody supershift experiments, 1 µl of anti-GFP or anti-polyhistidine (nonspecific control) antibody was added to binding reactions. In competition experiments, 10× and 100× unlabeled SL3 or mutated SL3 (mSL3) was added. (C) RNA binding capacity of GFP-L22 containing basic residue mutations or truncation of the amino-terminus (left panel) or with internal point mutations (right panel) was tested in RNA EMSA reactions, as described above. Amounts of each protein lysate used in binding reaction were determined by normalizing the level of expression of each mutated L22 construct to the level of GFP-L22 in 5 µg total protein lysate. Abbreviations used are: FP = free probe, NS = nonspecific, E = endogenous, GFP-L22 = all specific shifts generated with wild-type or mutated GFP-L22 proteins, SS = supershift.

To determine if the amino acid substitutions and truncations altered the ability of L22 to bind RNA, each mutated L22 protein was tested for its ability to bind EBER-1 RNA via electrophoretic mobility shift assay (EMSA). EBER-1 was chosen as the target RNA for this assay as its interaction with L22 has been well characterized and is of high affinity [19], [25]–[27]. Full-length EBER-1, in addition to interacting with L22 as demonstrated in Fig. 1B, interacts with several other cellular proteins, making interpretation of results somewhat complex [30]–[32]. Consequently, to evaluate the specific interaction between L22 and EBER-1, we chose to use an RNA oligonucleotide corresponding to stem-loop III (SL3) of EBER-1 which to date has been shown to interact only with L22. As shown in Fig. 3A, a concentration-dependent mobility shift of SL3 probe was observed when increasing amounts of protein lysate derived from cells expressing GFP-L22 were added to binding reactions (lanes 3–5). This mobility shift was not seen using lysate containing GFP alone (lane 2), even at concentrations exceeding 20 µg of protein lysate (data not shown). An additional faster migrating complex was also observed in all protein-containing reactions (lanes 2–5, designated “E”) and likely corresponds to a complex containing endogenous L22. To confirm that GFP-L22 was present within the slower mobility complexes formed with SL3, we performed antibody supershift assays using anti-GFP antibody to alter the mobility of GFP-L22 containing complexes. As demonstrated in Fig. 3B, while addition of anti-GFP antibody did result in a supershifted complex (lane 3, designated “SS”), addition of a nonspecific control anti-polyhistidine antibody did not alter the mobility of the shifted complex (lane 4). The overall reduction in intensity of signal observed in reactions containing anti-polyhistidine antibody may be the result of nuclease contamination of the antibody solution, however, we do not believe that this interfered with our ability to visualize a supershifted complex had there been one as longer exposures showed no evidence of any effect on the mobility of the observed complexes. Additional confirmation of binding specificity was obtained from competition experiments in which the wild-type, but not a mutated, SL3 oligonucleotide effectively competed with probe for L22 binding (compare 100× SL3 with 100× mSL3, Fig. 3B). Having established the specificity of the interaction between GFP-L22 and SL3, we next tested the RNA binding capacity of each mutated L22 protein. As depicted in Fig. 3C, truncation of the nine N-terminal amino acids (Δ N9, left panel), as well as specific mutation of lysines 13–16 (m13–16, right panel), had no effect on the ability of L22 to bind EBER-1 RNA. By contrast, mutations introduced into basic amino acid clusters located at residues 80–84 and 88–93 completely abolished RNA binding (right panel). Together, these results demonstrate that residues within the N-terminal region are not required for RNA binding and establish that basic amino acid residues located between 80–93 constitute the primary RNA-binding domain of L22.

**Figure 4:**
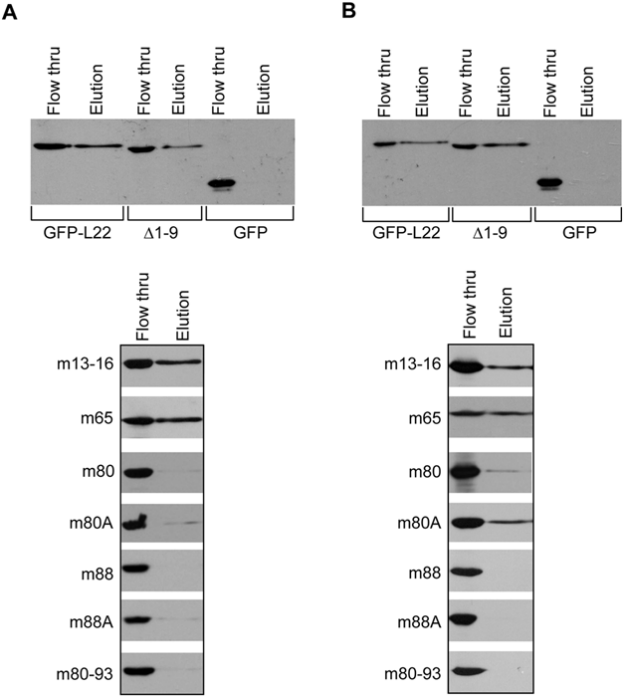
Mutation of residues 80–93 eliminates binding of L22 to multiple RNA substrates. The capacity of mutated L22 proteins to bind to EBER-1 (A) and 28S rRNA (B) was determined by specific capture of proteins on biotinylated RNAs immobilized on streptavidin magnetic beads. Protein lysates were generated from transiently transfected 293T cells and normalized for expression relative to wild-type GFP-L22. Total protein was incubated with 100 pmoles of biotinylated RNA and complexes were captured on streptavidin magnetic bead columns. Column flow-thru and eluate were subjected to SDS-PAGE and analyzed for GFP-L22 proteins by immunoblot using anti-GFP antibody. Protein lysate from cells transfected with GFP alone was used as a control for nonspecific binding to beads or RNA.

To obtain independent confirmation of our EMSA data and evaluate the requirements for L22 to bind 28S rRNA, we established a magnetic bead RNA-protein binding assay. In this assay, full-length EBER-1 or 28S rRNA stem-loop 7 (SL7) transcripts generated by *in vitro* transcription were annealed to biotinylated DNA oligonucleotides complementary to the 3′ end of each transcript. These biotinylated nucleic acid complexes were incubated with protein lysates and bound complexes were captured on streptavidin magnetic beads and analyzed by immunoblotting. As shown in Fig. 4, GFP-L22 and Δ1–9 were captured on the magnetic bead column in the presence of both EBER-1 (4A) and 28S rRNA (4B), as indicated by the band present in column eluates. GFP alone was unable to bind either RNA and was consequently not seen in column eluates. In agreement with the results presented in Fig. 3, m13–16 was found in the eluate in the presence of EBER-1, as was m65, demonstrating that both proteins bound EBER-1. Furthermore, as expected, mutation of basic residues 80–93 eliminated binding of L22 to EBER-1 (Fig. 4A, lower panels). Occasionally, very weak bands for these proteins were observed in column eluates. However, given the strength of this binding relative to that seen with wild-type and other mutated proteins and the results of our EMSA experiments, this is likely attributable to nonspecific interactions with the beads or RNA or possibly very weak affinity for the RNA. Following assay validation, we next evaluated the ability of these proteins to bind 28S rRNA (Fig. 4B, lower panels). As expected, m13–16 and m65 were captured in this assay. By contrast, m88, m88A and m80–93 were not found in the eluate, clearly demonstrating that basic residues 88–93 are absolutely critical for L22 to bind both EBER-1 and 28S rRNA. Somewhat surprisingly, m80 and m80A were present in the eluate, although m80 appeared to bind less well than m80A. Nevertheless, taken together with the EBER-1 data, it is likely that residues 80–84 contribute to the binding affinity of L22 for both RNAs, although perhaps to a lesser extent for 28s rRNA.

### Loss of RNA binding capacity alters the subcellular localization of L22

**Figure 5:**
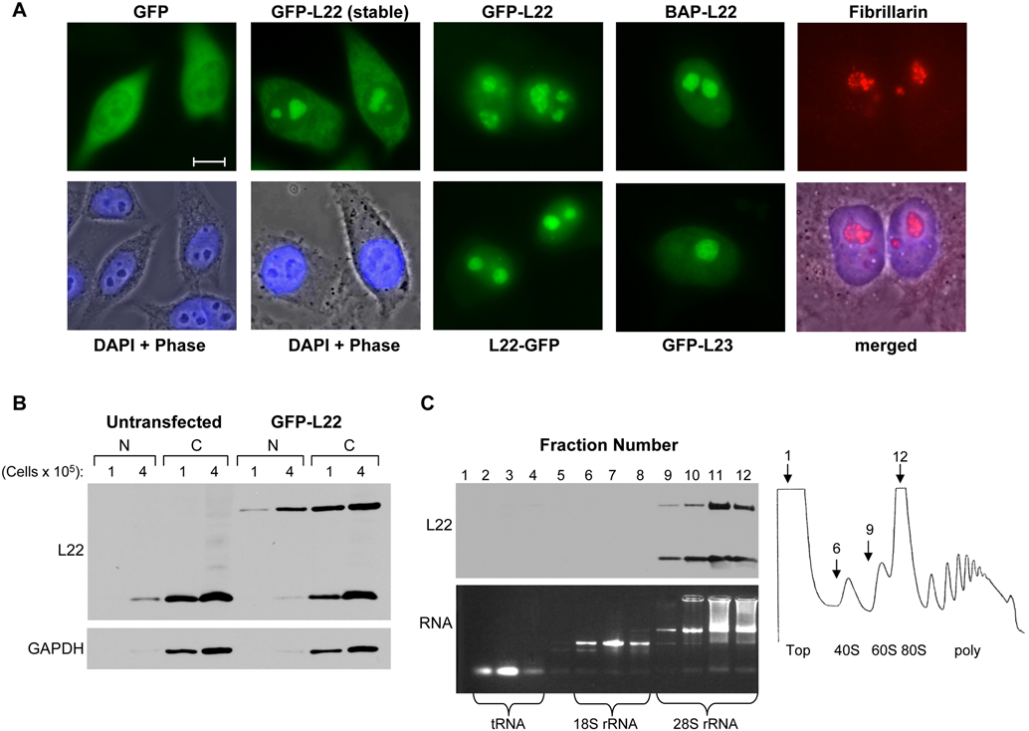
GFP-L22 is localized to nucleoli and incorporated into ribosomes. (A) The subcellular localization of wild-type L22 fusion proteins and control proteins was analyzed by fluorescence microscopy following expression in 293T and HeLa cells. For transient expression, cells grown on coverslips were transfected with 2 µg of the indicated expression construct, fixed after 48 hrs and visualized using a Zeiss Axiovert inverted fluorescence microscope. HeLa-L22 cells stably express GFP-L22. BAP-L22 expression was visualized in transiently transfected HeLa cells following staining with Alexa Fluor 488-conjugated streptavidin. Fibrillarin served as an endogenous nucleolar marker and was detected in HeLa cells using anti-fibrillarin antibody and Alexa-conjugated secondary antibody. All coverslips were mounted in Vectashield plus DAPI. Bar equals 10 µm. (B) Nuclear (N) and cytoplasmic (C) fractions from untransfected and GFP-L22 transfected 293T cells were analyzed by immunblot using anti-L22 and anti-GAPDH antibodies. Extract from the indicated number of cells was analyzed. Following detection of L22, blots were stripped of antibody and reprobed for GAPDH which served as a control for cytoplasmic contamination of nuclear extracts. (C) Localization of endogenous L22 and GFP-L22 in HeLa-L22 cells was assessed by sucrose density gradient analysis. Ribosome-containing lysates were separated on a 10–50% w/v sucrose gradient and 0.5 ml fractions were collected from the top of the gradient. The protein and RNA content of each fraction was analyzed by western blot and agarose gel electrophoresis, respectively. Total RNA was visualized by ethidium bromide. Polysome profiles were recorded during fraction collection at 260 nm. The ribosomal subunit composition of each peak is indicated along with fraction numbers corresponding to the first and last fraction collected (1 and 12) as well as the start of collection of the 40S (fraction 6) and 60S (fraction 9) peaks.

Several studies have established a link between the ability of certain proteins to bind rRNA and the nucleolar targeting and retention of these proteins (reviewed in: [5], [33], [34]). Given the finding that L22 binds 28S rRNA, we pursued the hypothesis that the interaction of L22 with 28S rRNA is essential for nucleolar localization of L22. To validate our system, we first evaluated the subcellular localization of unfused GFP, GFP-L22 (N-term), L22-GFP (C-term), BAP-L22, GFP-L23, and fibrillarin (an endogenous marker for nucleoli). While expression of GFP alone resulted in diffuse nuclear and cytoplasmic fluorescence, L22 tagged at either termini with GFP or BAP was localized to nucleoli (Fig. 5A). Of note, L22 proteins expressed transiently in 293T and HeLa cells typically displayed abundant nucleolar but little cytoplasmic fluorescence, whereas stably expressed GFP-L22 was observed in nucleoli and in the cytoplasm (Fig. 5A). We attribute this to the high level of L22 expressed in transiently transfected cells, resulting in intense nucleolar fluorescence which obscures the more diffuse GFP fluorescence in the cytoplasm. This conclusion is supported by the readily observable cytoplasmic fluorescence in stably expressing cells where GFP-L22 is expressed at lower levels and by the finding that a similar localization pattern is observed for GFP-L23 transiently expressed in 293T cells (Fig. 5A). Furthermore, when cells transiently expressing GFP-L22 were fractionated into nuclear (N) and cytoplasmic (C) fractions, GFP-L22 (upper band) was found in both fractions at levels equivalent to those seen for endogenous L22, (lower band) supporting the conclusion that GFP-L22 is abundantly present in the cytoplasm (Fig. 5B). Additionally, expression of GFP-L22 did not alter the localization of endogenous L22 as the fractionation pattern of L22 is equivalent in untransfected cells. To confirm that the inclusion of a GFP tag on L22 does not interfere with the ability of GFP-L22 to be incorporated into ribosomes, we performed sucrose density gradient analyses on cellular extracts from cells stably expressing GFP-L22 (Fig. 5C). The majority of GFP-L22 (upper band) was concentrated in fractions 9–12 which, as illustrated by ethidium bromide staining of electrophoresed RNA and by the polysome profile shown in Fig. 5C, also contain 28S rRNA and correspond to 60S ribosomal subunits. This fractionation pattern is equivalent to that seen with endogenous L22 (lower band) in these cells. Thus, we conclude that, despite the addition of a GFP tag, GFP-L22 is efficiently targeted to nucleoli and incorporated into cytoplasmic ribosomes.

**Figure 6:**
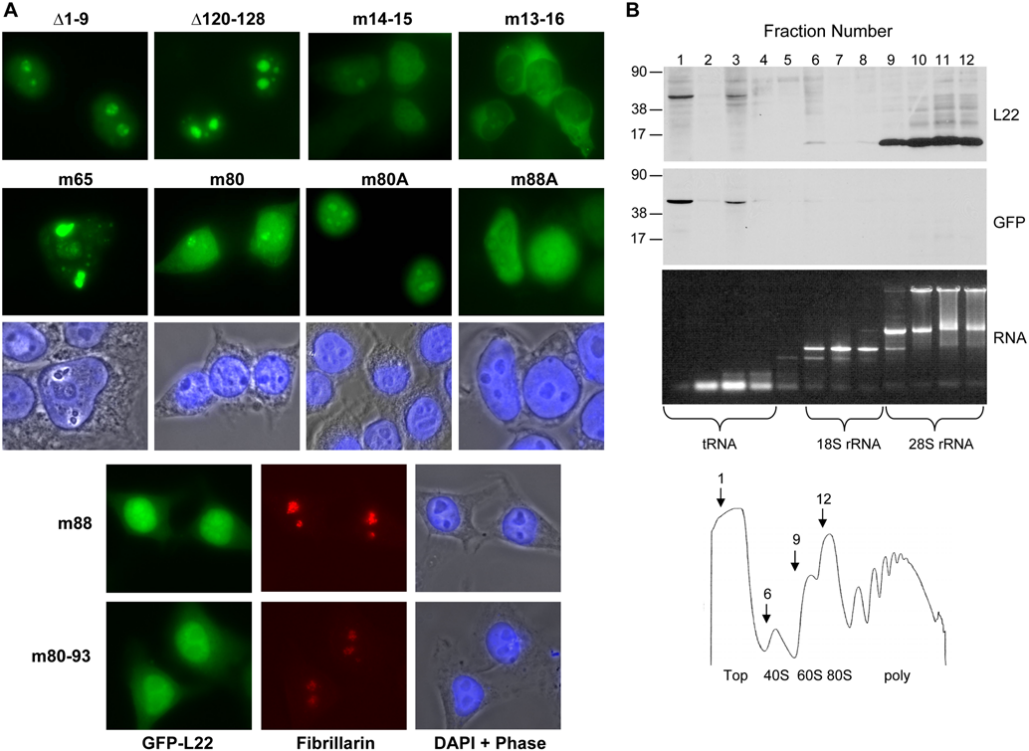
Mutation of residues 80–93 alters the subcellular localization of L22 and prevents incorporation into ribosomes. (A) The subcellular localization of mutated GFP-L22 proteins was analyzed by fluorescence microscopy following transient expression in 293T cells as described in Fig. 5A. (B) Incorporation of m88 into ribosomes was analyzed by sucrose density gradient analysis of extracts generated from 293T cells engineered to stably express m88, as described in Fig. 5B. Migration of molecular weight standards (in kDa) is indicated to the left of the blots. Following detection of L22 (15 kDa) and m88 (43 kDa) with anti-L22 antibody, the blot was stripped of antibody and reprobed with anti-GFP antibody to confirm that the 43 kDa bands present in fractions 1–3 were in fact GFP-tagged L22.

Having established that the addition of GFP does not alter the localization of wild-type L22 or its ability to be incorporated into ribosomes and transported to the cytoplasm, we next evaluated our panel of L22 mutations. As expected, given our hypothesis that RNA binding and nucleolar localization are correlated, Δ1–9 which is capable of binding 28S rRNA was found to be localized to nucleoli (Fig. 6A). An additional protein, Δ120–128, which is also capable of binding 28S rRNA (data not shown), was found to be similarly localized. Next, we investigated the consequence of internal point mutations on L22 localization. A previous study demonstrated that nuclear import of L22 depends on a classical nuclear localization signal consisting of a string of four lysine residues (13–16) preceded by a glycine residue [29]. In agreement with this, while mutation of only two lysine residues in this sequence (m14–15) resulted in increased nucleoplasmic and reduced nucleolar fluorescence relative to wild-type L22, mutation of all four lysine residues (m13–16) resulted in retention of L22 in the cytoplasm (Fig. 6A). Having defined the requisite sequences for nuclear entry of L22, we next evaluated the amino acid requirement for nucleolar targeting and retention. In agreement with our hypothesis, mutations which eliminated RNA binding also resulted in either exclusion of L22 from nucleoli or lack of retention of L22 in nucleoli (Fig. 6A: m88, m88A and m80–93). Furthermore, sucrose density gradient fractionation of a 293T cell line engineered to stably express m88 (Fig. 6B) revealed that the majority of m88 was found at the top of the gradient (fractions 1–3), as demonstrated by western blot analysis using both anti-L22 and anti-GFP antibodies (Fig. 6B, upper and middle panels). This fractionation pattern is distinct from that seen with endogenous L22 and wild-type GFP-L22 (Fig. 6B, fractions 9–12, top panel) in that unlike these proteins, m88 did not co-fractionate with 28S rRNA (found in fractions 9–12) and was not incorporated into ribosomal subunits. Consistent with our hypothesis, proteins with mutations in residues 80–84 (m80, m80A), which maintained some residual RNA binding capacity (Fig. 4B), also showed an intermediate pattern of localization in which L22 was mainly relocalized to the nucleoplasm with a small fraction of L22 retained in nucleoli. m65, a protein which is capable of binding RNA, showed two distinct patterns of localization. While a fraction of m65 localizes to nucleoli, a significant percent is found in dense precipitates within nuclei in a subpopulation of cells (Fig. 6A). As discussed above, transfection of m65 results in lower levels of protein expression relative to the other L22 constructs and may be somewhat cytotoxic in the subpopulation of cells containing these aggregates. These fluorescence localization and fractionation studies, together with the results of our RNA-binding studies, provide strong support for our hypothesis that interaction of L22 with 28S rRNA is a key determinant of nucleolar localization of L22.

## Discussion

Previous studies investigating the interaction between L22 and RNA have focused on defining the specific nucleotides required for L22 binding [19], [25]–[27]. Here, we have focused instead on defining the amino acid residues of L22 that contribute to its RNA binding capacity using two RNAs that match the binding site consensus previously defined by Dobbelstein and Shenk [25]. Our data establish that basic amino acids spanning 88–93 are critical for RNA binding by L22 and support a role for a second cluster of basic amino acids, 80–84, in high affinity RNA binding (Figs. 3 and 4). These amino acids were also found to be critical for proper targeting of L22 to the nucleolus (Fig. 6) thereby linking RNA binding and nucleolar localization.

Using a biotin-avidin affinity assay to confirm the *in vivo* interaction between L22 and the RNAs used in our subsequent *in vitro* binding assays, we observed strong binding of L22 to EBER-1 and binding, albeit significantly weaker, to 28S rRNA (Fig. 1). Binding of full-length 28S rRNA by L22 likely occurs in an ordered cascade with multiple additional ribosomal proteins and assembly factors. Once incorporated into a mature ribosomal subunit, the biotinylated BAP tag of L22 may no longer be easily accessible to the streptavidin beads used in this assay, resulting in the observed weak binding. However, less efficient binding of L22 to 28S rRNA relative to EBER-1 has been previously reported [25]. To identify the RNA-binding domain of L22, we evaluated a number of amino acid residues for their contribution to RNA binding *in vitro* (Fig. 2A). We focused primarily on internal clusters of basic residues but also evaluated amino acids 1–9. These N-terminal amino acids were chosen based on a previous report in which deletion of these residues, along with the eight C-terminal residues, prevented incorporation of L22 into 60S ribosomal subunits [29]. As truncation of only the C-terminal residues did not prevent incorporation, the authors predicted that the N-terminal residues might play a role in rRNA binding. Our data does not support this initial prediction as Δ1–9 did not differ from full-length L22 in its RNA binding capacity (Figs. 3 and 4). We instead found that amino acids 88–93 were absolutely required for binding of L22 to both EBER-1 and 28S rRNA SL7. Further, mutation of basic amino acids 80–84 eliminated binding of EBER-1 and reduced, but did not completely eliminate, binding of 28S rRNA (Fig. 4). Our data suggest that EBER-1 makes contact with amino acids 80–84 as well as 88–93 whereas the contacts for SL7 are primarily between amino acids 88–93. As we only used SL7, which represents a small portion of a much larger molecule, this leaves open the possibility that additional nucleotide sequences of 28S rRNA not included in our assay interact directly with residues 80–84. SELEX experiments in which two nucleotide sequences of 28S rRNA in addition to SL7 were bound by L22 strongly support this idea [25]. As no structural information for L22 is currently available, it is difficult to discern whether this newly identified RNA-binding domain conforms to any known RNA-binding motif. Studies have shown, however, that the RNA-binding domains of many ribosomal and nucleolar proteins appear to be coincident with and have the characteristics of nucleolar localization domains [14], [16], [35]. In light of this, we investigated whether the RNA-binding domain of L22 is critical for its nucleolar localization.

In agreement with Shu-Nu, et al., our results reveal that amino acids 88–93 are necessary for the nucleolar localization of L22 [29]. In addition, amino acids 80–84 were found to contribute significantly to nucleolar localization (Fig. 6A). A compact nucleolar localization signal (NOS) consisting of a nucleolar targeting sequence coincident with a nuclear localization signal (NLS) has been previously defined [16]. This compact NOS was shown to be sufficient for targeting a reporter protein to the nucleolus. Such compact NOSs are found in a number of ribosomal proteins including S25, S7 and L31 [7], [10], [11], [16]. Other ribosomal proteins, such as S19 and S6, require additional sequences for nucleolar accumulation to occur [8], [9]. To determine whether amino acids 80–93 function as a compact NOS, we evaluated the ability of a peptide containing only these residues to direct GFP to the nucleolus. This peptide failed to target GFP to the nucleolus demonstrating that amino acids 80–93 are not sufficient to function as a compact NOS, at least in the context of GFP (data not shown). In agreement with Shu-Nu, et al., we found instead that the NLS of L22 is contained within amino acids 13–16 (Fig. 6A) [29]. In addition to the NLS located near the N-terminus, Shu-Nu, et al. also proposed a role for the N-terminal and acidic C-terminal domains in mediating the subcellular localization of L22 [29]. We assessed the localization of L22 proteins truncated at either the N- or C- terminus and found that truncation did not alter the localization of L22 (Fig. 6A: Δ1–9 and Δ120–128; data not shown). It should be noted that our constructs and experimental design differ from those of Shu-Nu et al. in that FLAG-tagged proteins and anti-FLAG immunofluorescence were used by this group. We constructed and analyzed the localization of multiple epitope-tagged full-length and Δ1–9 L22 construct using immunofluorescence and obtained variable (different localizations within a population of cells) and inconsistent results. We attribute this to either problems associated with fixation of cells or access of antibody to L22 incorporated in nucleoli. We experience no such difficulties with either our GFP-tagged (N- or C-terminal) or BAP-tagged proteins.

Our finding that amino acids 80–93 mediate both RNA binding and nucleolar localization suggests that the nucleolar accumulation of L22 is a consequence of its binding 28S rRNA. Such a link between RNA binding and nucleolar accumulation is not novel. In fact, many nucleolar proteins have been shown to contain RNA-binding sites coincident with sequences critical for nucleolar localization. These include Nop25, in which RNA binding is a requisite for nucleolar localization, nucleolin, in which nucleolar import is contingent upon two RNA-binding domains and p120, in which the arginine-rich RNA-binding domain is coincident with the NOS [35]–[37]. Furthermore, binding of rRNA was found to mediate nucleolar localization of a number of ribosomal proteins including S25, L5, and L7a [14], [16], [17]. It is not surprising that sequences classified as NOSs are often discovered to contain RNA binding activity since NOSs are generally rich in basic residues, a feature amenable to binding negatively charged nucleic acids [7], [12], [14], [16]. These findings together with our data lead us to conclude that the nucleolar localization of L22 is likely mediated by its interaction with specific sequences of 28S rRNA.

As binding of both EBER-1 and 28S rRNA SL7 require the same basic residues of L22 for binding, our data suggest that binding of RNAs matching the consensus derived by Dobbelstein and Shenk is mutually exclusive. This is substantiated by the findings of Toczski et al. demonstrating that association of L22 with EBER-1 or the ribosome is mutually exclusive, implying competition between RNA ligands [26]. Since rRNA binding is linked to nucleolar accumulation, it might be speculated that EBER-1 evolved to bind L22 during virus infection and prevent accumulation of L22 in nucleoli. Indeed, it has been shown that L22 is relocalized from nucleoli to the nucleoplasm in EBV-infected cells [26]. Furthermore, other viral RNAs and proteins, such as HCV 3′X RNA, HVP-1 RNA and HSV ICP4 and ICP22 proteins, have been shown to interact with L22, raising the possibility that L22 may play a central role during viral infection [18], [24], [38], [39]. This raises the question of whether these viruses sequester L22 to prevent or modulate some cellular function of L22 or whether L22 plays an active role in virus replication. As the normal role of L22 remains essentially undefined it is difficult to answer this question. L22 is not required for basal translation *in vitro* nor is deletion of L22 in mice a lethal phenotype [20], [22]. Interestingly, L22-deficient mice showed a selective block in αβ T cell development while γδ T cells were unaffected. Together, this suggests that L22 performs extra-ribosomal or regulatory functions as have been described for multiple eukaryotic ribosomal proteins (reviewed in: [40]). L22 is also known to interact with human telomerase RNA (hTR) [23]. As a fraction of hTR is known to localize to nucleoli, this is not unexpected [41], [42]. Given that hTR and EBER-1 would likely be in competition for L22 binding in an EBV-infected cell, one might predict that telomerase activity would be altered in these cells if EBER-1 sequesters L22 as L22 traffics through the nucleoplasm to reach the nucleolus. Furthermore, use of mutants of L22 which have lost the capacity to bind RNA would be predicted to alter telomerase activity as well as potentially impact the nucleolar localization of hTR. Current and future studies are aimed at characterizing the consequences of relocalization of L22 during viral infection and tumorigenesis in an effort to understand the role of L22 during these as well as normal cellular processes.

## Materials and Methods

### Cell culture

293T and HeLa cells were maintained in Dulbecco's modified Eagle's medium (Mediatech) supplemented with 4.5 g of glucose per liter, 2 mM L-glutamine, 10% fetal bovine serum and 1% penicillin/streptomycin. HeLa cells stably expressing GFP-L22 (HeLa-L22) were generated by FuGENE (Roche) transfection of HeLa cells with pcDNA3.1-GFP-L22 and subsequent selection of transfectants in media containing 600 µg G418 per ml. 293T cells stably expressing m88 (293-m88) were generated by FuGENE (Roche) co-transfection of 293T cells with pcDNA3.1-GFP-m88 and a hygromycin resistance plasmid, followed by subsequent selection of transfectants in media containing 600 µg G418 and 200 µg hygromycin per ml.

### Plasmids and site-directed mutagenesis

L22 cDNA was generated by reverse transcription PCR using total RNA isolated from Akata Burkitt lymphoma cells. Primers used in amplification were: 5′-ATATGGATCCCCATGGCTCCTGT-3′ and 5′-GATCGAATTCCACTGACGAGATACAAGG-3′. Amplified cDNA was digested with *Bam*HI and *Eco*RI, then cloned into digested pcDNA3 to generate pcDNA3-L22. This base construct was used in subsequent PCR reactions to generate additional wild-type and mutated L22 expression constructs. PCR amplified DNA fragments were cloned into pcDNA3.1/NT-GFP-TOPO (Invitrogen) to generate N-terminal GFP fusion constructs. To generate L22-GFP (C-terminal GFP fusion), L22 sequence was PCR amplified from pcDNA3-L22 using primers which incorporated 5′ *Bam*HI and 3′ *Eco*RI sites. GFP sequence was PCR amplified from pcDNA3.1/NT-GFP using primers which incorporated 5′ *Eco*RI and 3′ *Xba*I sites. Digested PCR products were cloned into *Bam*HI and *Xba*I digested pCR3.1, creating L22-GFP. The Δ1–9 deletion construct was generated by PCR using the 5′ primer: 5′-AAGGGAGGCAAAAAAAAGAAGCAAGTTCTG-3′ along with the wild-type 3′ primer. Substitution mutants were generated via QuikChange site-directed mutagenesis (Stratagene) followed by PCR amplification and TOPO cloning. Specific amino acids altered to generate each mutation are depicted in Fig. 2. GFP-L23 was a gift from H. Lu (Indiana University School of Medicine). To generate the base BAP vector (pCR3.1BAP), complementary oligonucleotides containing the biotin acceptor peptide (BAP) sequence and the tobacco etch virus protease (TEV) cleavage sites were annealed (5′-AGCTTATGAGCGGACTCAACGACATTTTCGAGGCCCAAAAGATCGAATGGCACGAAGAGAATCTGTACTTTCAGG-3′ and 5′-GATCCCTGAAAGTACAGATTCTCTTCGTGCCATTCGATCTTTTGGGCCTCGAAAATGTCGTTGAGTCCGCTCATA-3′) and ligated to *Hind*III and *Bam*HI digested pCR3.1 (Invitrogen). L22 sequence was PCR amplified from pcDNA3-L22 using primers which incorporated 5′ *Bam*HI and 3′ *Xba*I sites and cloned into *Bam*HI and *Xba*I digested pCR3.1BAP to create pCR3.1BAP-L22. To generate pSG5-EBERs, the entire EBV *Eco*RI-J genomic restriction fragment, containing both EBER-1 and EBER-2 and their transcriptional regulatory elements, was ligated into *Eco*RI digested pSG5 (Stratagene). pTER-EBER-1 and pTER-EBER-2 were generated by PCR amplification of the EBER-1 or EBER-2 coding sequence using primers incorporating 5′ *Bam*HI and 3′ *Hind*III sites and cloned into *Bgl*II and *Hind*III digested pTER [43]. All plasmids were sequence verified and tested for correct expression as described below.

### Transfection and immunoblotting

To verify correct expression of GFP-tagged wild-type and mutated L22, 293T cells were transiently transfected with 2 µg of each plasmid using FuGENE6 (Roche) according to the manufacturer's suggested protocol. 48 hrs post-transfection, cells were harvested, washed in PBS and resuspended in lysis buffer (50 mM Tris-Cl [pH 8.0], 150 mM NaCl, 1 mM EDTA, 1% Triton X-100, Complete™ protease inhibitor cocktail [Roche]) followed by incubation on ice for 10 min. Insoluble material was removed by centrifugation at 12,000× g for 10 min. The protein concentration of the supernatant was determined by the Bradford method. 30 µg total protein was fractionated on a 12% SDS-PAGE gel, transferred onto Immobilon-P membrane (Millipore) and processed using anti-GFP antibody (JL8 BD Living Colors, Clontech).

### RNA-binding assays

For MACS binding assays, linear templates for *in vitro* transcription were generated by introducing a T7 promoter upstream of the coding sequence of EBER-1 and stem-loop 7 (SL7) of 28S rRNA via PCR using the following primers: EBER: 5′-TAATACGACTCACTATAGGGACCTACGCTGCCC-3′ and 5′-AAAACATGCGGACCACCAGCT-3′; 28S: 5′ TAATACGACTCACTATAGGGAGTCGGGTTGCTTGGGAA-3′ and 5′-CGCCCTCTTGAACTCTC-3′. *In vitro* transcription was performed using the MEGAshortscript T7 kit (Ambion) according to the manufacturer's instructions. RNA-protein interaction was assessed via streptavidin-labeled magnetic bead assay (MACS, Miltenyi Biotec) in which EBER-1 RNA generated by *in vitro* transcription was annealed to a biotinylated oligonucleotide (5′-bio-AAAACATGCGGACCACCAGCTGGTACT-3′) complementary to the 3′ end of EBER-1. 28S rRNA SL7 transcript was likewise annealed (5′-bio-CGCCCTCTTCTTCTCTCTCTTCAAAGT-3′). These biotinylated RNA-DNA hybrids were incubated with protein lysate isolated from transiently transfected 293T cells and bound complexes were captured on streptavidin-labeled magnetic beads using µMACS columns. Column flow-thru, washes and eluted bound protein were isolated and analyzed by immunoblot, as described above.

For EMSA analyses, an RNA oligonucleotide corresponding to nucleotides 56 to 87 of stem-loop III of EBER-1 was end-labeled with [γ-^32^P] ATP using T4 polynucleotide kinase. Unincorporated nucleotides were removed using NucAway columns (Ambion). Prior to use, the probe was boiled for 3 min. and immediately placed on ice for 3 min. 10 µl binding reactions containing 10 mM HEPES (pH 8.0), 50 mM KCl, 1 mM EDTA, 0.1 mM DTT, 0.1% Triton X-100, 2.5% glycerol, 1 µg yeast tRNA and protein lysate were incubated on ice 10 min. prior to addition of 0.05 pmoles probe and incubation on ice for an additional 20 min. Protein-RNA complexes were resolved by electrophoresis in non-denaturing 8% polyacrylamide gels run at 4°C in 0.5× TBE or TTE (National Diagnostics). Following electrophoresis, gels were dried and processed by autoradiography. Antibody supershifts were used to assess binding specificity by adding 1 µl of antibody to binding reactions 10 min. after the addition of probe. Competition experiments utilized 10× and 100× molar excess of either unlabeled EBER-1 SL3 or mutated SL3 (5′-CACCCGGCCAUGGUACAAGGCCAUGGUGGUGA-3′) RNA oligonucleotides added 10 min. prior to the addition of probe.

To generate *in vivo* biotinylated BAP-L22 in the presence of EBERs for biotin-avidin affinity assays, 293T cells were co-transfected with 1 µg each pBirA biotin ligase (provided by Dr. Adam Geballe), pCR3.1BAP-L22 and EBER expression constructs in the presence of 25 µM biotin. 48 hrs post-transfection, cells were UV irradiated on ice for 4.5 min. (254 nM) and lysed in 500 µl NET-N (50 mM Tris [pH 8.0], 150 mM NaCl, 1 mM EDTA, 0.5% NP-40, Complete™ protease inhibitor cocktail [Roche]). Following centrifugation to pellet cell debris, lysates were mixed with 100 µl immobilized avidin beads (Pierce) and rocked at 4°C for 1 hour. Beads were pelleted, separated from the supernatant, washed 5× with NET-N and resuspended in 300 µl elution buffer (100 mM Tris [pH 8.0], 150 mM NaCl, 1% SDS, 12.5 mM EDTA) followed by heating at 65°C for 15 min. RNA from the supernatant and bound fractions was extracted with phenol-chloroform and ethanol precipitated. Total bound RNA and 2.5 µg supernatant RNA were loaded onto a 1.2% agarose-2.2 M formaldehyde gel and processed by northern hybridization using standard protocols. Verification of biotinylated BAP-L22 isolation was accomplished as above except that beads were resuspended in 2× SDS loading dye and electrophoresed on a tricine peptide gel followed by immunoblot analysis using anti-L22 (BD Transduction Laboratories) and HRP-conjugated streptavidin (Vector Labs).

### Fluorescence localization

293T cells were seeded into 60 mm tissue culture dishes containing coverslips and transiently transfected with GFP-tagged wild-type or mutated L22 expression constructs using FuGENE6 (Roche) as described above. 48 hrs post-transfection, coverslips were washed in PBS and fixed 10 min. in cold methanol followed by 10 min. in cold acetone. HeLa-L22 cells were seeded as above and fixed in 4% paraformaldehyde for 10 min. followed by 0.25% Triton X-100 for 10 min. BAP-L22 was visualized by staining with Alexa Fluor 488-conjugated streptavidin (Molecular Probes). Fibrillarin was detected using anti-fibrillarin antibody (Abcam Ab4566) and Alexa Fluor 568-conjugated anti-mouse secondary antibody. Coverslips were mounted in Vectashield plus DAPI (Vector Laboratories) and fluorescence visualized using a Zeiss Axiovert inverted fluorescence microscope.

### Isolation of nuclear and cytoplasmic extracts

Extracts from untransfected and transiently GFP-L22 transfected 293T cells were generated using the NE-PER® kit (Pierce) as per the manufacturers' instructions. Extracts from 1–4×10^5^ cells were electrophoresed on a tricine peptide gel and immunoblotted using anti-L22 antibody as described above. The integrity of the nuclear and cytoplasmic fractions was assessed by immunoblotting using anti-GAPDH antibody (Imgenex).

### Sucrose density gradient analyses

Ribosome containing lysates were prepared using a protocol adapted from Arava, et al., 2003 [44]. Cells were treated with 0.1 mg/ml cycloheximide for 3–5 min at 37°C, pelleted by centrifugation, washed in PBS and resuspended at a density of 3–8×10^7^ cells per ml in ribosome lysis buffer (20 mM Tris, [pH 8.0], 140 mM KCl, 1.5 mM MgCl_2_, 0.5 mM DTT, 1% Triton X-100, 0.1 mg/ml cycloheximide). Lysates were homogenized on ice using a dounce homogenizer and tight pestle. Following centrifugation for 10 minutes at 12,000× g, lysates were overlaid onto a 10–50% (w/v) sucrose gradient prepared by overlaying 10% buffered sucrose (20 mM Tris [pH 8.0], 140 mM KCl, 5 mM MgCl_2_, 0.5 mM DTT, 0.1 mg/ml cycloheximide) onto 50% buffered sucrose followed by horizontal diffusion for 3 hours. Gradients were centrifuged at 35K RPM in an SW41 rotor for 160 min at 4°C. 0.5 ml fractions were collected using an Isco fractionator and Foxy Jr. fraction collector. Fractions were divided into 2 aliquots for subsequent RNA and protein analysis.

Total RNA from gradient fractions was prepared by phenol-chloroform extraction and ethanol precipitation. RNA was fractionated by electrophoresis through a 1.2% agarose gel and visualized by ethidium bromide staining. Protein was prepared from gradient fractions by TCA precipitation using Na-deoxycholate (125 ug/ml) and trichloroacetic acid (6%). Following incubation on ice for 15 min and centrifugation, pellets were washed 2× with cold acetone, resuspended in 1× SDS loading buffer and loaded onto tricine peptide gels. Proteins were transferred onto Immobilon-P membrane (Millipore) and immunoblotted using an enhanced chemiluminescent detection system (HyGLO, Denville Scientific). Primary antibodies utilized were: anti-L22 (BD Transduction Labs) and anti-GFP (JL8 BD Living Colors, Clontech). Immunoreactive proteins were detected using HRP-conjugated secondary antibodies. For reprobing, membranes were stripped of antibody in stripping buffer (62.5 mM Tris-Cl [pH 6.8], 100 mM 2-mercaptoethanol, 2% SDS) for 30 min at 50°C.
